# A Novel Concept of Combined High-Level-Laser Treatment and Transcutaneous Photobiomodulation Therapy Utilisation in Orthodontic Periodontal Interface Management

**DOI:** 10.3390/s22062263

**Published:** 2022-03-15

**Authors:** Reem Hanna, Magdalena Pawelczyk-Madalińska, Tudor Sălăgean, Mircea Emil Nap, Ioana Roxana Bordea, Stefano Benedicenti

**Affiliations:** 1 Department of Surgical Sciences and Integrated Diagnostics, Laser Therapy Centre, University of Genoa, Viale Benedetto XV, 6, 16132 Genoa, Italy; m.m.fandent@gmail.com (M.P.-M.); stefano.benedicenti@unige.it (S.B.); 2 Department of Oral Surgery, King’s College Hospital NHS Foundation Trust, London SE5 9RS, UK; 3 Department of Periodontology, Pomeranian Medical University, Rybacka 1, 70-204 Szczecin, Poland; 4 FAN-DENT Centrum Stomatologii i Periodontologii, Juliusza Słowackiego 71/2, 80-257 Gdańsk, Poland; 5 Department of Land Measurements and Exact Sciences, University of Agricultural Sciences and Veterinary Medicine Cluj Napoca, 400372 Cluj-Napoca, Romania; tudor.salagen@usamvcluj.ro; 6 Department of Oral Rehabilitation, “Iuliu Hațieganu” University of Medicine and Pharmacy Cluj-Napoca, 400012 Cluj-Napoca, Romania; roxana.bordea@ymail.com

**Keywords:** Er,Cr:YSGG, HLLT, gingivoplasty, LLLT, oral wound regeneration, PBM, photochemical, photothermal, surgical diode laser, upper midline frenectomy

## Abstract

This case report is aimed to demonstrate the synergetic effects of λ940 nm laser photobiomodulation (PBM) therapy in augmenting the advantages of high-level-laser treatment (HLLT)-mediated reaction orthodontic periodontal interface management. Materials and Methods: A 32-year-old female who presented with a persistent gummy smile of upper incisors and low upper midline frenum attachment post-orthodontic treatment, was seeking a better smile appearance. She had a history of delayed wound healing without underlying medical conditions; otherwise, she was fit and healthy. She underwent laser ablation of the upper midline frenum and gingivoplasty of the upper incisors region with λ940 nm and λ2780, respectively, as well as transcutaneous PBM therapy (λ940 nm) to accelerate wound healing. The laser protocols were as follows: λ2780 nm: power output—2 W, pulse width—60 μs, free running pulse (FRP), spot area—0.0016 cm^2^, pulse repetition rate—25 pulses per second (s), 80 mJ/pulse, 90 s, λ940 nm: 1.2 W, continuous wave (CW) emission mode, 300 μm, 60 s; whereas the adjunctive λ940 nm induced-PBM parameters were as follows: power output—1.4 W, CW—120 s, single application, spot area—2.8 cm^2^. An acceleration of the wound healing was observed on the 4th day of treatment with no immediate or post-operative complications. The results showed no functional or aesthetic relapses at a long-term follow-up of 6 months. The authors concluded that λ940 nm laser-PBM can provide a synergetic effect to HLLT in accelerating wound healing and offering a precision smile with minimal to none post-operative complications. It is safe and justifiable to utilise dual therapy over the conventional methods, which serves our patients’ needs in our daily practice and in various clinical indications. The concept and laser protocols of this clinical case report can pave the roadmap for future extensive studies.

**Highlights**

1. High-level-laser treatment (HLLT)-mediated reaction converged with surgical λ940 nm and λ2780 nm is effective in post-orthodontic soft tissue healing.
2. This clinical communication demonstrated the effectiveness of HLLT in upper midline frenectomy and upper gingivoplasty from aesthetic and functional standpoints.
3. A transcutaneous 940 nm PBM-laser can provide a synergetic effect, as an adjunct, to HLLT in accelerating wound healing and regeneration, which ultimately can optimise the clinical outcome and be utilised in daily practice.

## 1. Introduction

The contextual relationships between orthodontics and periodontology are diverse and complex. Gummy smile (healthy gingival overgrowth) correction has been a challenge for many orthodontists and periodontists [1]. An orthognathic surgery or botox could be options [2]; however, due to the associated post-operative complications, patients tend to consider alternative methods of treatment [3]. Additionally, upper midline frenum with low attachments has a great impact on patients from cosmetic and functional (pull syndrome) standpoints [4]. On these notes, conventional surgical interventions (electrosurgery and scalpel) have been considered the standard treatment modalities in the management of upper midline frenectomy and gingivoplasty due to surgical scalpel precision [5]. In these therapies there is: a lack of bloodless field, post-operative complications, surgical trauma, long surgical time, the requirement for a good mucosal anaesthesia, and healing with primary intention where sutures are needed [6,7,8] when compared to laser-assisted surgery, which has overcome these limitations due to its advantageous properties [8,9,10,11]. It is noteworthy that there are surgical laser drawbacks such as surgical precision and collateral thermal events, which can be minimised by employing the following fundamental parameters [12]: minimal therapeutic power output, correct emission mode, thermal relaxation time, cooling methods, a knowledge of the target biological properties, an experienced laser clinician, and a precision surgical laser tip [7].

**Figure 1:**
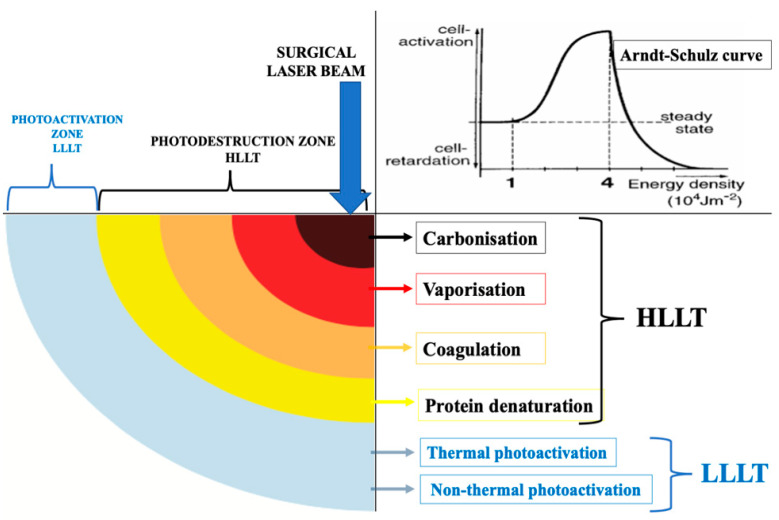
An illustration of the concept of the surgical laser beam profile where the photonic energy λ940 nm, λ2970 nm of its first zone (HLLT) is absorbed by haemoglobin (Hb) and water, respectively, which is subsequently transformed into thermal energy and then causes tissue destruction (tissue ablation), whereas the last zone of laser beam is LLLT (Modified) [13,15]. The top right graph is an “Arndt–Schulz curve”, illustrating the biphasic dose response measured in the difference in the integrated area under the curve of the time course of wound size compared to a no-treatment control, with different modes of cell reaction at different levels of energy density [17].

With regard to a complete healing time, there is controversy in the literature between conventional methods and surgical laser treatments of various wavelengths due to multifactorial reasons, including the chosen laser parameters [8]. Therefore, the utilisation of a high-level-laser treatment (HLLT)-mediated reaction of Er,Cr:YSGG (λ2780 nm) and surgical diode at λ940 nm results in tissue ablation with a peripheral zone of simultaneous low-level-laser therapy (LLLT) around the surgical site [13,14,15]. High-level-laser treatment (HLLT) is defined as high levels of incident laser power that are utilised to cause the photodestruction of a specific target tissue through a light–heat transduction process, inducing photothermal damage of varying degrees [16]. HLLT is used in many surgical fields. Figure 1 schematically illustrates the concept of simultaneous LLLT. An area of thermal and nonthermal photoactivation is produced simultaneously at the periphery of a high-powered surgical laser beam (HLLT) together with photodestructive reactions. Additionally, in Figure 1, a graph of the Arndt–Schulz curve illustrates the concept of the Gaussian beam profile of LLLT [17]. It is important to note that LLLT is a formal term for photobiomodulation therapy (PBMT).

**Figure 2:**
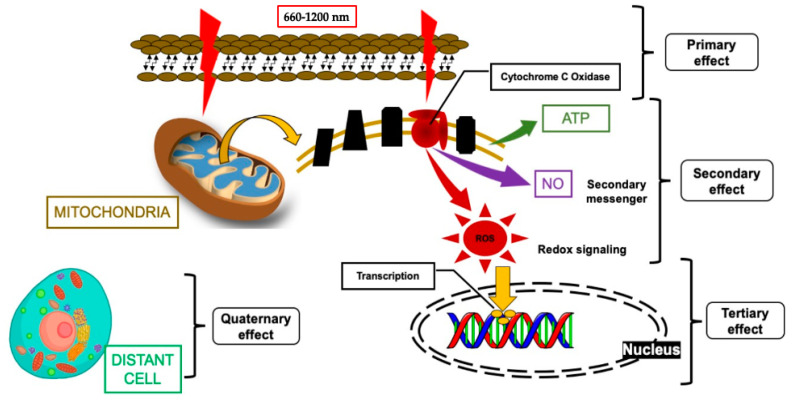
Modified schematic description of the of mechanism of action for PBM, highlighting its four effects: the primary effects (photonic energy absorption by cytochrome C Oxidase (CCO)), secondary effects (mitochondrion of ATP, NO, and ROS), tertiary effects (downstream of intracellular responses effects (mitochondrion of ATP, NO, and ROS), and quaternary effect (indirect/distant effects such as gene transcription and cellular signaling) [21]. Abbreviations: PBM, photobiomodulation; ROS, reactive oxygen species; NO, nitric oxide; ATP, adenosine triphosphate.

The effect of transcutaneous 940 nm-PBMT has a great impact in accelerating healing time and enhancing the healing process [18,19,20]. Figure 2 illustrates the mechanism of action of PBM that is based on the four following effects [21,22,23]: (1) the primary effect is the photonic energy of λ660–1200 nm, which are within the optical window for absorption by the cytochrome C oxidase (CCO) on the inner membrane of the mitochondria, resulting in cellular and molecular cascades [21]; (2) the secondary effect refers to the changes in adenosine triphosphate (ATP), nitric oxide (NO), and reactive oxygen species (ROS) [22], which are dependent on dose and redox states [23]; (3) the tertiary effect is related to the downstream biochemical effects [22], which are context- and cell-type-specific (direct or indirect way), affecting the cell membrane and nucleus, controlling the gene transcription and, subsequently, cell proliferation, migration, apoptosis, and inflammation [23]; and (4) the quaternary effects (distant, systemic effects) of PBM are associated with the tissues that have not absorbed the photonic energy, yet can still be affected indirectly by secretions of the cells that have absorbed the laser light [22]. Hence, the present case report is aimed to demonstrate the synergetic effect of λ940 nm-PBM in augmenting the advantages of λ2970 nm and surgical diode λ940 nm, in improving the smile-line with minimal post-operative complications, accelerating healing time and enhancing wound healing based on a long-term follow-up.

## 2. Materials and Methods

### 2.1. Study Design

An interventional clinical prospective communication evaluated the synergetic effect of 940 nm-PBM of the HLLT in accelerating oral wound healing and improving gummy smile by utilising HLLT (λ2970 nm and λ940 nm surgical lasers) and a PBM laser- λ940 nm. An informed written consent was obtained from the patient, confirming a full understanding of the proposed treatments, benefits, advantages, drawbacks, and alternative treatments. In addition, a written consent was obtained from the patient for scientific publication.

### 2.2. Study Participant and Case Description

**Figure 3:**
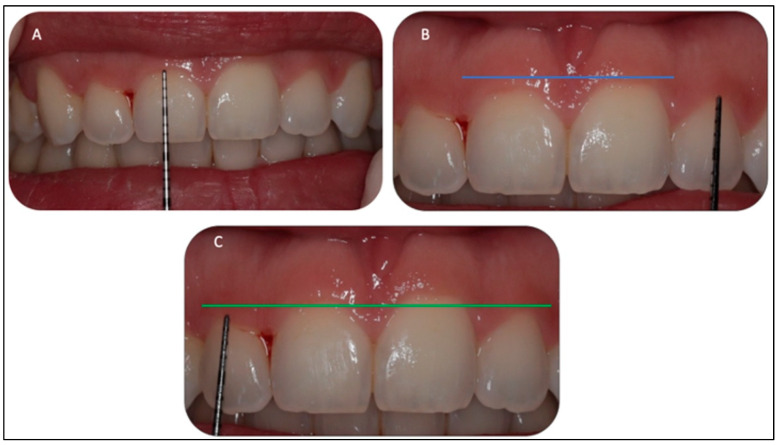
(**A–C**). Clinical assessment of #11, #12, #21, and #22 revealed gingival overgrowth with no periodontal pocket or inflammation. The biological width was determined by measuring the proposed ablative gingival tissues with a periodontal probe and marking the proposed zones with an orthodontic marker. In addition, it shows the pocket depth determination with the periodontal probe. Biologic width requires the probe to pierce the attachments so that the clinician can accurately measure the distance to the osseous crest. Evidence of pre-operative discrepancy in the level of the gingival margin of the #11, compared to the #21, is highlighted in blue photo (**B**) as well for # 12 and #22, as highlighted in green photo (**C**).

**Figure 4:**
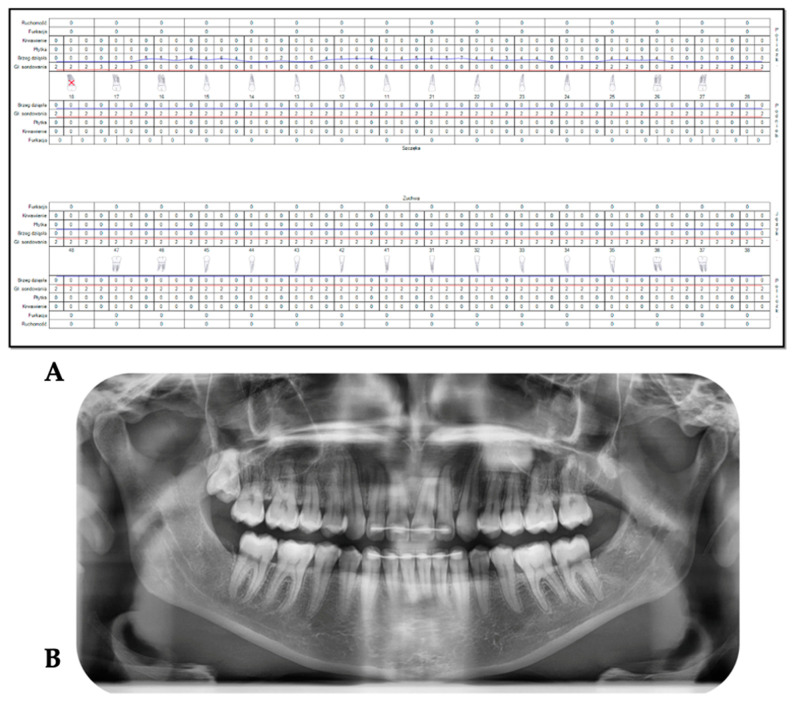
(**A**,**B**). Pre-operative clinical and radiographic investigations. Photo (**A**) (top) is the periodontal charting, showing no evidence of any abnormality. Photo (**B**) (bottom) shows no evidence of any hard tissue abnormalities on the orthopantomogram.

A fit and healthy 32-year-old female patient was referred to the Advanced Laser Clinic by her orthodontist on the 16 December 2019, three months after she completed her orthodontic treatment. The patient’s duration of orthodontic treatment was for two years to correct her gummy smile. However, residual excessive gingival display in the region of the upper incisors remained, representing level I according to the maxillary excess classification [24]. The orthodontist referred the patient for gingivoplasty of #11,#12,#21, and #22 [25] to improve the patient’s gummy smile and upper midline frenum to prevent gum recession of #11 and #21 (pull syndrome). The diagnosis was gingival overgrowth of a non-inflammatory nature that could be related to gingival margin, but did not achieve an adequate level during eruption. A low attachment of the upper midline frenum was the diagnosis, due to the anatomical variation in the frenum attachments’ insertions. The purpose of the frenectomy in this clinical case was to prevent pull syndrome. The patient’s main concern was her smile. The patient was fit and healthy, but reported one episode of delayed healing after a tooth extraction two years previous, in which investigations showed no underlying medical problems. Upon examination, all tooth sites showed no bleeding on probing and no evidence of mobility and calculus. However, a grade II gingival overgrowth of #11, #12, #21, and #22, including the dental papillae (Figure 3A–C) was observed. Figure 3B,C shows evidence of a discrepancy in the gingival zenith between #11 and #21, as well between #12 and #22, respectively, which were present prior to the orthodontic treatment, according to the patient. Figure 4A,B shows no periodontal abnormalities on orthopantomogram and periodontal charting. The upper midline frenum was a thick fibrotic Class II (gingival frenal attachment) according to the Kotlow Classifications [26]. The general level of oral hygiene was considered good.

### 2.3. Interventions

#### 2.3.1. Er,Cr:YSGG (λ2780 nm) Surgical Laser

**Figure 5:**
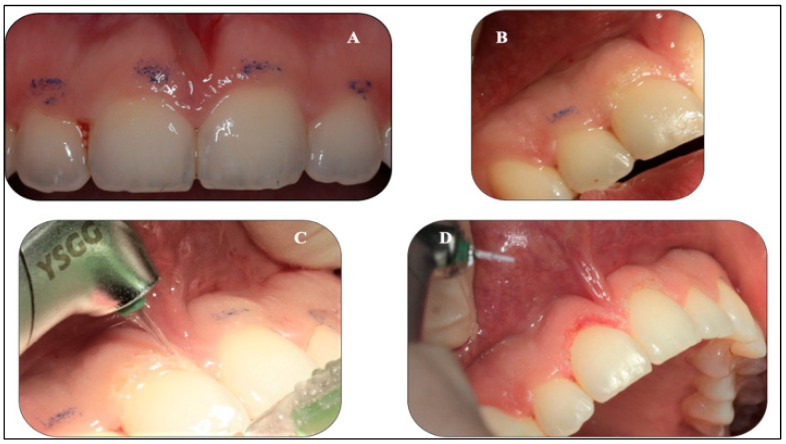
(**A**–**D**). Clinical photos (**A**,**B**) show the markings of the proposed gingival margin utilised by the orthodontic pencil based on the measurements, taking into consideration the biological width. The clinical photo (**C**) shows the angualtion of the Er,Cr:YSGG tip at 45° to follow the marked ginigval tissue. Clinical photo (**D**) shows an immediate gingivoplasty of #11.

The objectives in utilising λ2780 for gingivoplasty of #11, #12, #21, and #22 were as follows: (1) to ablate the gingival overgrowth tissue effectively while maintaining the biological width by measuring the proposed ablative gingival tissues with a periodontal probe and marking the areas with an orthodontic marker (Figure 5A,B), thus providing a proper gingival re-contouring that matched the rest of the teeth; (2) minimal post-operative complications; (3) optimal haemostasis; and (4) shallow penetration depth of approximately 300 μ and the pulsed emission provided by operator control during the procedure.

#### 2.3.2. λ940 nm Surgical Diode Laser

A λ940 nm surgical diode laser was objectively utilised to effectively excise the fibrotic upper midline frenum attachment and reallocate them to the correct position with minimal peri- and post-treatment complications. In relation to haemostasis, a diode laser is superior to Er,Cr:YSGG, as the photonic energy of λ940 nm is well absorbed in soft tissue haemoglobin [27], whereas λ2780 nm is highly absorbed by water (predominant chromophore) [28,29]. Therefore, λ940 nm is more suitable to ablate a thick fibrotic frenum.

#### 2.3.3. λ940 nm-PBM-Transcutaneous Approach

The objectives of λ940 nm laser-PBM utilisation were to accelerate the healing time, enhance wound healing, and provide analgesic and anti-inflammatory effects. This wavelength has a deep penetration depth, which is suitable to reach the target. Ultimately, PBM therapy is suitable in this clinical communication, as the patient has a history of a delay in wound healing without underlying reasons.

### 2.4. Outcomes Assessment Measures

A visual analogue scale (VAS) [30] was utilised to assess pain intensity and the clinical grading of wound healing assessment was based on the following descriptions [31]: Grade 1: sloughy; Grade 2: no granulation; Grade 3: granulation; Grade 4: re-epithelialisation; Grade 5: completely epithelialised.

### 2.5. Treatment Description

#### 2.5.1. Gingivoplasty Procedure

Buccal infiltration with a total of 1.2 mL of 2% lidocaine in 1:80,000 adrenaline was administered in the upper anterior teeth region away from the upper midline frenulum. Each tooth of the upper anterior teeth was measured twice and the gingivae were marked with the orthodontic pencil (Figure 5A,B), identifying the proposed new gingival margin. The MT4 tip of λ2780 was angled at 45° to the gingiva and followed the marked points to facilitate resection of the overgrown tissue (Figure 5C).

The total treatment duration was 90 s. The clinician has taken in consideration how the average potential power (2 W) might etch the enamel surface with the MT4 tip, so they have employed the following precautions and treatment techniques: (1) surgical loupes to improve the visibility of the treatment field; (2) the tip of MT4 was angled at 45° to the enamel surface during the ablation; (3) the last layer of the gingival tissue was removed with a periodontal curette to protect the enamel, despite knowing the penetration depth of λ2780 is approximately 300 microns (μ); (4) a clear plastic strip protection was used to avoid enamel destruction; (5) employed optimal parameters, including a free running pulse emission mode (thermal relaxation); and (6) employed cooling methods such as air and water.

The authors strongly emphasise the importance of taking the abovementioned operating and technical steps into consideration in order to achieve an optimal outcome. Table 1A shows wavelength specifications, laser parameters, and calculations. Figure 5D shows the new gingival margin of the #11 immediately post-treatment. After completion of the gingivoplasty, the upper midline frenum was checked for pull syndrome.

#### 2.5.2. Upper Midline Frenectomy Procedure

**Figure 6:**
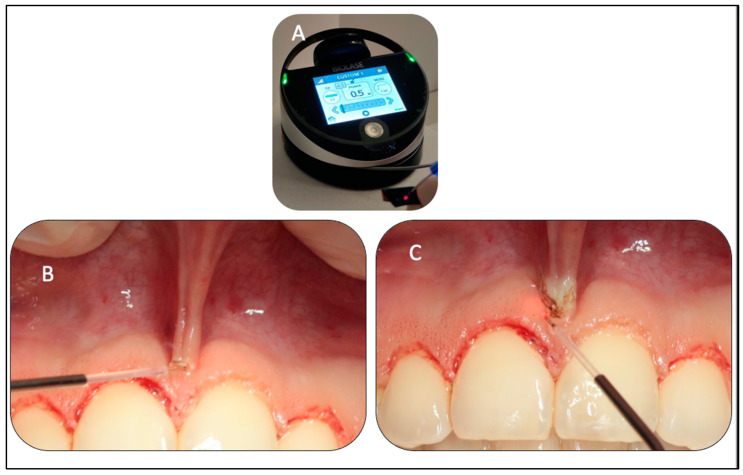
(**A**–**C**). Clinical photo (**A**) shows the tip initiation of the λ940 nm diode laser fibre using articulating paper, aiming to generate the maximum energy on its tip. Photo (**B**) is a peri-operative image, illustrating the upper midline frenum under stretch as well the light–tissue interaction, illustrating the initial ablation of the frenum attachments. Photo (**C**) is a peri-opearive image, showing the laser fiber direction at 90° to the frenum tissue and parallel to the alvoalr bone and away from the teeth positons.

The tip of the diode was initiated (Figure 6A). Table 1B shows the laser parameters and calculations employed for λ940 nm. The frenum was placed under tension to identify the profile of the muscle fibre insertion. The initiated fibre of λ940 nm was held perpendicular to the target tissue and parallel to the alveolus at 2–3 mm away from fixed gingival tissue, where an initial ablation was performed (Figure 6B). Subsequently, the ablation advanced to a depth where superficial muscle fibres were detached with no blanching or movement of gingival tissue of #11 and #21 observed (Figure 6C).

**Figure 7:**
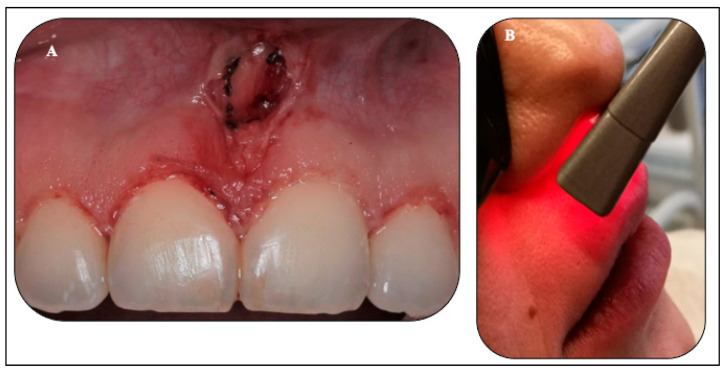
(**A**,**B**). Clinical photo (**A**) was taken immediately following laser treatments, illustrating the rhomboid shape of the new position of the upper midline and the gingival re-contouring of the upper incisor teeth. Photo (**B**) is an extra-oral application of λ940 nm laser-PBM in the region of the treated sites.

Figure 7A shows an immediate post-laser gingivoplasty of #11, #12, #21, and #22, displaying the rhomboid shape of the new upper midline frenum attachment. Any char on the tissue or fibre tip was removed with damp gauze in order to avoid self-initiation and overheating of the tissue. The treatment site was occasionally cooled down using damp gauze during the treatment, in order to avoid heat build-up at the treatment site by removing the first ablated tissue layer to allow better photonic energy penetration. Additionally, vacuum suction was used and directed to the treatment site as a source of cooling and laser plume removal.

#### 2.5.3. Transcutaneous λ940 nm Laser-PBM (Extraoral Approach)

Immediately after laser treatments, λ940 nm laser-PBM was irradiated extra-orally with a whitening handpiece of 2.8 cm^2^ spot size, where photonic energy was delivered transcutaneously over the upper lip area in the region of the treatment sites (Figure 7B) at power output of 1.4 W in a continuous emission mode (CW) for 120 s in a single application (Table 1C).

## 3. Results

The patient received post-operative instructions and review appointments scheduled at four-and 14-days post-treatment, as well at six-months post-operatively. The VAS [30] and clinical wound healing grading tools [31] were employed to evaluate pain intensity and wound healing grade, respectively.

**Figure 8:**
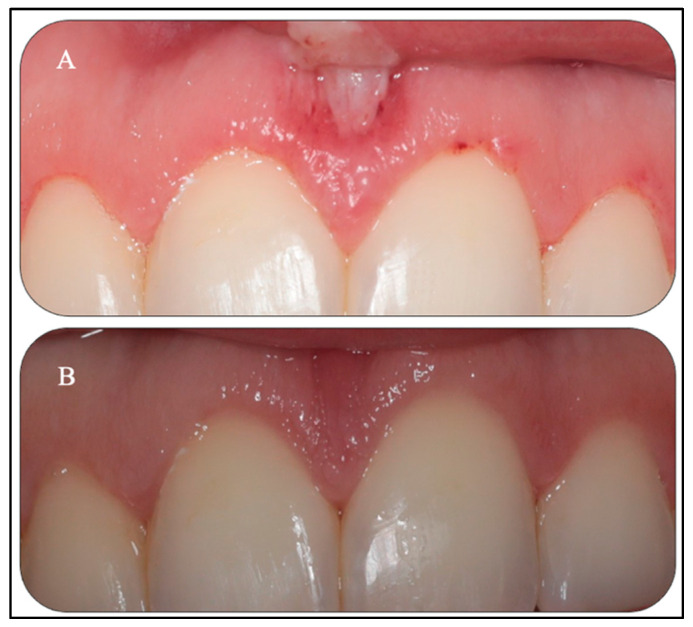
(**A**,**B**). Clinical photo (**A**) reveals the healing status at the fourth day post-laser treatments, where the healing status of marginal gingival tissues of the #11, #12,# 21, and # 22 was grade IV, and the upper midline frenum shows re-epithelisation (grade IV), indicating an acceleration of the healing process. Clinical photo (**B**) reveals a complete healing of the upper midline frenum (Garde V) and great wound healing progress towards complete wound healing of the gingival margin of #11, #21, #12, and #22 (grade IV) at two weeks post-treatments.

Figure 8A shows evidence of a band of re-epithelisation (Grade IV) of the frenum and a grade IV of healing status of gingival tissue of #11, #12, #21, and #22, at the fourth day post-laser treatments. Figure 8B shows a complete healing (Grade V) of the upper midline wound site and great wound healing progress towards complete wound healing of the gingival margin of #11, #21, #12, and #22 (Grade IV), at 14-days post-operatively, with a great smile improvement and no evidence of pull syndrome. The patient did not report any post-operative complications such as pain, oedema, and bleeding. Patient’s self-reporting revealed no pain, rating a zero score on the VAS post-operatively.

**Figure 9:**
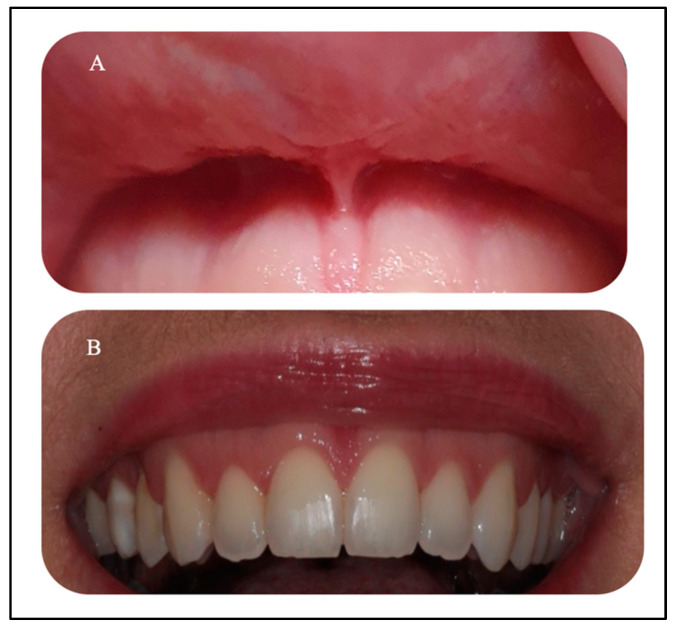
(**A**,**B**) Clinical photos of both laser treatments at six-months post-laser treatments. Photo (**A**) shows a complete healing of the upper midline frenum with new frenum attachment without evidence of functional relapse. Photo (**B**) shows a complete healing of the gingival tissue with a great improvement in the gingival margins of the upper incisor teeth.

**Figure 10:**
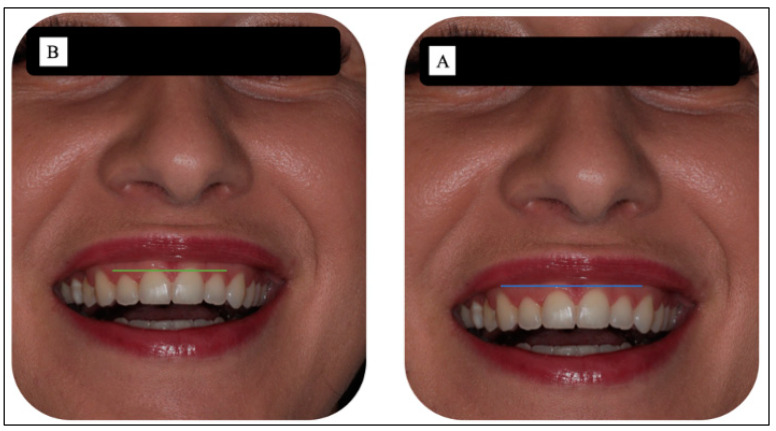
(**A**,**B**). At six-months post-laser treatment, photo (**A**) illustrates the level of the zeniths (blue line) of the gingival tissue of #11 and #21, whereas photo (**B**) illustrates the level of the zeniths (green line) of the gingival tissue of #12 and #22. The photos show an improvement in the clinical outcomes from an aesthetic standpoint.

The long-term results at six months (Figure 9A,B) were in keeping with the objectives of the original treatment plan. In Figure 9A, the upper midline frenectomy procedure shows uneventful frenal functional attachment with no evidence of relapse. Equally, in Figure 9B, the gingival re-contouring of the upper incisor teeth procedure has been uneventful, with a great improvement of her initial gummy smile (patient’s main concern) and enhanced the patients’ natural zeniths’ discrepancies, which were prior to the laser treatment and even before the patient’s orthodontic treatment (Figure 10A,B). In this context, a great improvement was noted, rather than reporting a relapse. This patient was very satisfied with the clinical outcomes.

**Figure 11:**
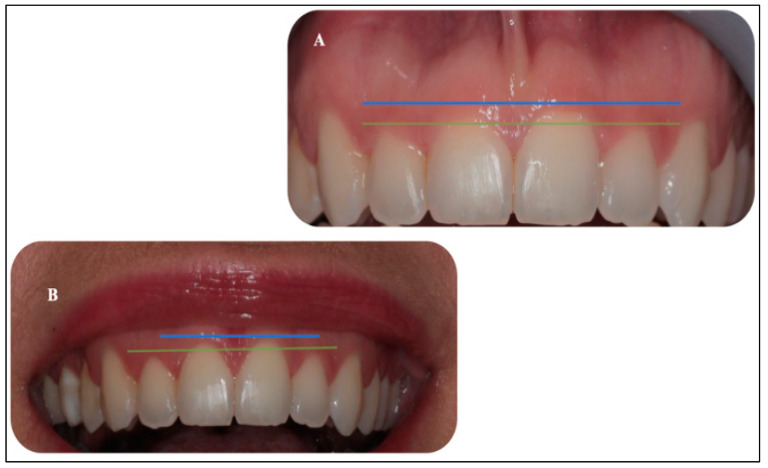
(**A,B**) Illustration of the difference between the gingival overgrowth of the # 11, #12, #21, and #22 pre-operatively photo (**A**) and the (photo (**B**)) clinical outcome of the smile line at six-months post-laser gingivoplasty. Blue and green lines show the zeniths of the gingival tissue of #11 and #21, and #12 and #22, respectively. Photo (**B**) shows a great improvement in the gingival zeniths of upper incisors at six months, compared to those taken pre-operatively photo (**A**).

**Figure 12:**
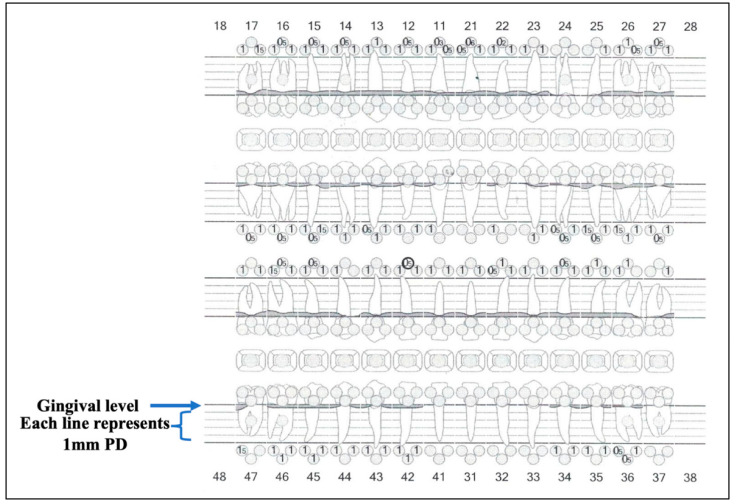
A periodontal chart at six-months post-laser treatments shows a healthy biological width and clinical attachment, as there is no evidence of gum recession and the pocket depth (PD) of the #11, #12, #21, and #22 ranged between 0–1 millimetres (mm). The PD was measured in mm at six points for each tooth (labial/buccal mesial, labial/buccal central, labial/buccal distal, palatal/lingual mesial, palatal/lingual central, and palatal/lingual distal). The empty circle represents a PD of 0 mm.

Figure 10A,B shows the optimal outcome of the gingivoplasty with patient’s satisfaction six-months post-operatively. Figure 10A shows the level of the zeniths (blue line) of the gingival tissue of #11 and #21, whereas photo B illustrates the level of the zeniths (green line) of #12 and #22 gingival margins. The photos highlight the clinical outcomes from an aesthetic standpoint. Additionally, Figure 11A,B illustrates the great improvement in patient’s smile line at six-months post-laser treatment (Photo B), compared to the pre-operatively (Photo A). Moreover, Figure 12 shows a healthy biological width and clinical attachment at six months post-treatment, with no evidence of gingival recession and a PD of #11, #21, #12, and #22 that ranged between 0–1.

## 4. Discussion

The aim of this case report study was to evaluate the synergistic effects of λ940 nm laser-PBM therapy in augmenting the effects of HLLT-mediated reaction orthodontic periodontal interface management. This study has shown that HLLT of two wavelengths (λ940 nm and λ2780 nm) in surgical oral soft tissue management related to post-orthodontic treatment was a useful treatment tool from aesthetic and functional standpoints. However, the utilisation of a λ940 nm diode laser-PBM has proven to have a synergetic effect in shortening the healing time, accelerating wound healing (Grade V) and re-epithelisation (Grade IV) with no post-operative complication at the fourth day post-laser treatment. The long-term follow-up at two weeks and six months have proven no evidence of functional and aesthetic relapses.

Many wavelengths in the range of infra-red (IR), mid-IR, and far IR of the electromagnetic spectrum have been employed for surgical oral soft tissues conditions [12,32,33,34]. HLLT has been utilised in many surgical oral soft tissues related to orthodontic treatment [35]. It is important to report that there is controversy in the literature regarding the complete healing time among various wavelengths of HLLT [36,37]. On this note, λ940 nm laser-PBM that is within the optical window for an optimal outcome, where the photonic energy is absorbed by cytochrome C oxidase and, subsequently, a cascade of cellular and molecular activities occurs, ultimately leading to positive results in accelerating the healing process and alleviating pain [18,20], which was observed in the present case report.

Photonic energy of shorter wavelengths (500–1000 nm) are readily absorbed in pigmented tissue and haemoglobin (Hb) through a photothermal phenomenon, whereas a λ940 nm surgical diode has a high affinity for melanin and less interaction with Hb. Hence, in our communication, the photonic energy of λ940 nm was absorbed by melanin of the of the mucosal tissue of the upper midline frenum [38], which led to the generation of an appropriate heat over 100 °C (photothermal phenomenon) [38] to ablate its fibrotic attachments with a satisfactory haemostasis in a shortened time of 60 s, resulting in a substantial clinical outcome. The longer wavelengths are more interactive with water and hydroxyapatite, with the largest absorption peak for water being just below 3000 nm, which is at the Er:YAG wavelength [39]. In λ2780 nm, the predominant chromophore is water and there is a lower affinity to pigmented tissue; however, as 75–90% of the keratinised and non-keratinised gingival tissue is water [27,40], our chosen photoablative parameters led to a peak absorption of the photonic energy with minimal thermal damage. This was supported by the histological analysis conducted by Cercadillo-Ibarguren et al. in 2016, which showed that the width of the thermal effect was 9–15 μm at 1–4 W where an Er,Cr:YSGG laser irradiated the incisions of porcine oral mucosa with water spray, in contrast to 10–60μm at 1–2 W without water spray [41].

It is noteworthy that the transmission event of laser energy is directly through the tissue with no effect on the target tissue, which is the inverse of absorption. However, this effect is highly dependent on the laser wavelength. Water, for example, is relatively transparent to the shorter wavelengths such as diodes, whereas tissue fluids readily absorb photonic energy of the erbium family and carbon dioxide (CO_2_) lasers at the outer surface. Hence, there is a little energy transmitted to adjacent tissues [38].

Importantly, a high-water absorption is inversely proportional to the reflection, scattering, and transmission of IR radiation, which accounts for minimal dispersed energy and thermal adverse effects in the surrounding tissues, not exceeding a range of 50 μm [3].

It is noteworthy to indicate that the utilisation of an average power output of 2 W delivered in a free running pulse (FRP), where the pulse width was 60 micro seconds (µs), showed to be effective in providing good gingival margin precision without jeopardising the biological width of the upper incisor teeth and minimising undesired thermal side effects [42]. Additionally, the shallow penetration depth (300 µ) of λ2780 nm gives the clinician control during the ablation process [39]. Moreover, λ2780 nm property at our chosen parameters (Table 1A) offers a selective soft tissue ablation, keeping the integrity of hard tissues intact.

## 5. Limitations of the Study and Future Perspectives

The empirical results reported herein should be considered in the light of some limitations. Our formulated PBM laser and HLLT protocols were based on the available case series [43] and the authors’ expertise and routine practice. Thereby, RCT studies utilising our protocols are warranted. This will prove the validity of our transcutaneous laser-PBM and HLLT protocols. Our results, for the first time, demonstrate the synergetic effects of transcutaneous 940 nm-PBM in augmenting HLLT of two wavelengths (λ940 nm and λ2780 nm) and the advantages in improving aesthetical and functional outcomes. Therefore, a large sample of subjects is necessary for future clinical studies to validate our clinical outcomes.

## 6. Conclusions

Our results, for this first time, demonstrated the concept of the synergetic effects of λ940 nm laser-PBM in augmenting the advantages of HLLT (surgical λ940 nm and λ2780 nm) in orthodontic-periodontal interface management. This was demonstrated from an aesthetic and functional standpoint by achieving a precision smile with minimal to none post-operative complications and accelerating oral wound healing, respectively. From a scientific community standpoint, our results are fundamentally useful for a clinician to utilise our HLLT and PBM protocols and parameters in various clinical applications to improve patients’ outcomes and experiences. Furthermore, the results of the present case report are very relevant to scholars and investigators to conduct extensive studies to validate our laser protocols and novel concept of a PBM synergetic effect in augmenting the efficacy of HLLT in orthodontic-periodontal interface management.

## Figures and Tables

**Table 1:** (**A**–**C**). (**A**) Description of the utilised laser and the adjustable and calculated laser operating parameters of λ2780 nm. (**B**) Description of the laser utilised and the adjustable and calculated laser operating parameters of λ940 nm. (**C**) Description of the λ940 nm laser-PBM utilised and the adjustable and calculated laser operating parameters. Abbreviations: Er,Cr:YSGG, Erbium Chromium: Yttrium Scandium Gallium Garnet; CW, continuous emission mode.

(A) Er,Cr:YSGG (λ2780 nm)		(B) Surgical Diode λ940 nm		(C) λ940 nm Laser-PBM	
**Laser**	Waterlase I PLUS 2.0 (Biolease)	**Laser**	Epix X (Biolease)	**Laser**	Epix X (Biolease)
**Wavelength**	2780 nm	**Wavelength**	940 nm	**Wavelength**	940 nm
**Co-axial aiming beam**	635 nm laser, 1 mW max.	**Co-axial aiming beam**	625–670 nm Class 2 (red)	**Co-axial aiming beam**	625–670 nm, Class 2 (red)
**Energy distribution**	Gaussian	**Energy distribution**	Gaussian	**Energy** **distribution**	Gaussian
**Delivery system**	Optical Fibre Cable, Hand-piece	**Delivery** **system**	Quartz fibre optic E3–7	**Delivery** **system**	Whitening contour handpiece
**Beam diameter**	0.0016 cm^2^	**Beam diameter**	0.0016 cm^2^	**Beam diameter**	rectangular
**Tip-to-tissue** **distance**	Contact (0 mm)	**Emission mode**	CW	**Emission mode**	CW
**Spot diameter at tissue**	0.0400 cm	**Tip-to-tissue distance**	Contact	**Tip-to-tissue distance**	5 mm
**Spot area at tissue**	0.0013 cm^2^	**Tip area**	0.0007 cm^2^	**Tip area**	2.8 cm^2^
**Emission mode**	Free running pulse	**Spot diameter at tissue**	0.0300 cm	**Spot diameter at tissue**	0.86 cm^2^
**Pulse width**	60 µs (H mode)	**Spot area at** **tissue**	0.0007 cm^2^	**Power output**	1.4 W
**Peak power**	1333 Watts (W)	**Energy delivery**	Initiated	**Spot area at tissue**	3.42 cm^2^
**Average power**	2 W	**Average power**	1.2 Watts	**treatment area**	7 cm^2^
**Average power density**	1592 W/cm^2^	**Fibre diameter**	300 µm	**Fibre diameter**	8 × 35 mm
**Pulse repetition rate**	25 PPS	**Bean divergence**	8°	**Total energy**	168 J
**Energy per pulse**	80 mJ	**Total energy**	72 J	**Energy density with** **movement**	196.5 J/cm^2^
**Total energy**	180 J	**Energy density with movement**	199.6 J/cm^2^	**Average power** **density**	24 W/cm^2^
**Average energy** **density**	143 238 J/cm^2^	**Average power density**	1698 W/cm^2^	**Duration of** **treatment**	120 s
**Peak power density**	1061 033 W/cm^2^	**Length** **of treatment**	60 s	**Number of** **application**	Single application
**Average power density**	1592 W/cm^2^	**Speed of** **movement**	2 mm/s		
**Water irrigation**	15 mL/min				
**Amount of air**	20 mL/min				
**Length of treatment**	90 s				
**Speed of movement**	1 mm/s				

## Data Availability

The data is contained within the article.
